# Effect of Cryodiluent and Time of Glycerol Addition on Cryopreservation and In Vitro Fertilization of Domestic Cat Epididymal Spermatozoa

**DOI:** 10.3390/ani15121680

**Published:** 2025-06-06

**Authors:** Natalia Gañán, Raquel González, Ana Sanchez-Rodriguez, Eduardo R. S. Roldan

**Affiliations:** Department of Biodiversity and Evolutionary Biology, Museo Nacional de Ciencias Naturales (CSIC), 28006 Madrid, Spain; natalia.gannan@gmail.com (N.G.); rrraquelgh2@hotmail.com (R.G.); anasanchez@mncn.csic.es (A.S.-R.)

**Keywords:** sperm, cryopreservation, cryodiluent, glycerol, in vitro fertilization, swim-up, pentoxifylline

## Abstract

Sperm cryopreservation and assisted reproduction are powerful tools for the conservation of endangered species. The domestic cat has been a useful model for studying wild felid reproductive biology due to the limited availability of endangered individuals for experimental research. Here, we investigate the effect of cryodiluents (TEST vs. Biladyl) and the timing of glycerol addition (before vs. after refrigeration, in one vs. three steps, respectively) on post-thaw sperm quality (motility, acrosome integrity) and their subsequent in vitro fertilization (IVF) ability with homologous oocytes. The results showed no statistically significant differences in sperm traits when samples were cryopreserved in TEST or Biladyl, or when glycerol was added in one or three steps. Motile sperm and intact acrosomes were significantly correlated before and after cryopreservation, indicating consistent relationships in fresh and thawed samples. The use of Biladyl significantly reduced IVF rates after cryopreservation compared to fresh sperm. Cryopreservation in TEST led to IVF rates that were not significantly different from those of fresh sperm. Using swim-up after thawing, or adding 1 mM pentoxifylline, did not enhance IVF results. Overall, a TEST cryodiluent with 4% glycerol added in one step is a reliable option for preserving epididymal cat spermatozoa.

## Simple Summary

This study explores methods to freeze cat sperm for conservation, using domestic cats as a model for endangered wild felids. We tested two freezing solutions (TEST and Biladyl), different glycerol addition protocols, and treatments to prepare spermatozoa for in vitro fertilization. No significant differences in sperm quality (motility, acrosome integrity) were found between the two freezing solutions or the glycerol addition methods. Percentages of total sperm motility and acrosome integrity were positively associated before and after freezing. Fertilization rates were lower in Biladyl-treated spermatozoa compared to fresh sperm, whereas sperm cryopreserved in TEST showed fertilization rates similar to those of fresh sperm. In conclusion, freezing cat sperm using TEST diluent, and adding glycerol in a single step (which may facilitate work in the field), results in reliable fertilization outcomes.

## 1. Introduction

The role of sperm cryopreservation in safeguarding endangered species has received widespread recognition over the past few decades [1,2,3,4,5,6]. The development of these protocols necessitates experimental investigations to understand how various factors involved in the freeze–thawing process impact spermatozoa across different species. For endangered species, such research can be exceptionally challenging, which has led to the use of experimental models. These models, typically domestic species closely related to the endangered ones, provide a practical approach to gathering sufficient samples and conducting necessary experiments. This allows researchers to refine techniques and subsequently tailor them to the target wildlife species [3,7,8]. In the field of sperm cryobiology, the domestic cat (*Felis catus*) has emerged as the primary model for wild felids [3,7,9,10], not only as an accessible source of gametes but also for the development of freezing protocols for epididymal sperm, particularly in the case of post-mortem recovery of non-domestic feline individuals.

Many studies have examined factors involved in the cryopreservation process of domestic cat spermatozoa that may affect sperm parameters. In particular, attention has been placed on how these factors influence sperm quality and functionality after thawing in order to achieve high fertilization rates via in vitro fertilization [11,12,13,14,15], intracytoplasmic sperm injection [16,17,18] or artificial insemination [19,20].

A fundamental factor in the cryopreservation process is the composition of the cryodiluent, particularly with regard to salts, energy sources, buffer, pH, and osmolality. The physicochemical changes that occur during cryopreservation are, at least in part, due to the concentration of the components in the aqueous solution in which the spermatozoa are refrigerated and frozen. Since the success of assisted reproductive technology programs heavily depends on the specific extender solutions and cryoprotective agents used for sperm cryopreservation, essential for male fertility preservation, the cryoprotectant deserves special attention. This includes its nature (penetrating or non-penetrating), the time and the temperature at which the spermatozoa are exposed to it [21]. Cryoprotective agents can be added or removed in steps, i.e., gradually increasing or decreasing their concentration in the freezing medium to reduce osmotic stress on the cell [22], or added or removed all at once, which reduces exposure time to the cryoprotectant that may be toxic [5]. The gradual, stepwise addition or removal of cryoprotectants minimizes motility loss and membrane disruption [23]. Other studies have analyzed the effect of osmotic changes, oxidative stress and thermal injury during cryopreservation [23,24,25,26,27,28]. For feline sperm preservation, glycerol and egg yolk are the two most common components used in semen extenders [28]. Glycerol has been incorporated into cryopreservation solutions at concentrations ranging from 3 to 8% [25,29], with 4–5% being the most commonly used [30,31]. Previous studies have examined the timing and method of glycerol addition to cryodiluents with varied results [5,32,33,34], highlighting the need for a thorough understanding of these aspects in order to design suitable protocols for new species, ensuring optimal outcomes and minimizing the negative effects of cryopreservation.

It is important to evaluate the fertilizing capacity of sperm subjected to cryopreservation. In vitro fertilization using intact conspecific oocytes represents a valuable laboratory method for assessing the reproductive potential of fresh or cryopreserved sperm samples. This test is a critical component of sperm cryopreservation research as it enables evaluation of the sequential aspects of sperm function required for fertilization, from capacitation to syngamy [35], as well as the sperm ability to generate embryos. In cats, there has been a steady increase in the development of IVF, which facilitates its use in laboratory settings [36].

Due to the decrease in motility and the proportion of spermatozoa with intact acrosomes, that occur as a result of cryopreservation, systems for processing sperm samples after thawing have been developed to favor fertilization in various assisted reproduction contexts. One such method is swim-up, which selects progressively motile spermatozoa and removes glycerol, egg yolk, cellular debris and non-motile spermatozoa. In this technique, fresh or frozen sperm are incubated to allow motile spermatozoa to swim upward through the culture medium, separating them from cell debris and less motile spermatozoa that remain at the bottom of the tube. This simple method recovers a higher proportion of motile and morphologically normal spermatozoa compared to simple dilution [12,37,38].

Another approach to improving motility in cryopreserved sperm involves the use of sperm motility stimulants such as pentoxifylline. This compound inhibits phosphodiesterases and stimulates motility without compromising sperm viability or lifespan [39]. Pentoxifylline increases intracellular cyclic adenosine-3′,5′-monophosphate (cAMP) levels by inhibiting its degradation, which is associated with the induction of sperm capacitation and hyperactivation [39,40]. It can be effective in treating male infertility by improving sperm motility and overall sperm quality [40]. In humans, pentoxifylline has been used to initiate motility in epididymal and testicular sperm for ICSI [41], and it has been reported to preserve sperm membrane integrity [42].

The aim of this study was to evaluate two key factors involved in sperm cryopreservation, using the domestic cat as a model species, to refine freezing protocols suitable for field application. We carried out three experimental series to assess: (1) the efficacy of sperm cryopreservation using two cryodiluents (TEST and Byladyl) and two glycerol addition protocols to TEST diluent, (2) the IVF capacity of cryopreserved spermatozoa, and (3) the effects of swim-up or pentoxifylline addition on post-thaw motility enhancement.

## 2. Materials and Methods

### 2.1. Animals and Reagents

Sperm samples were collected from the epididymides of domestic cats (*n* = 90) following routine orchiectomies performed at veterinary clinics and animal shelters in Madrid. This study did not require ethical review or approval, as it involved the analysis of epididymal sperm cells from cats that had been neutered either as part of a community spay program or at the owner’s request. The neutering procedures were not conducted specifically for the purposes of this research.

Reagents and ready-made solutions were purchased from Sigma-Merck (Madrid, Spain) or Invitrogen Technologies (Barcelona, Spain), unless stated otherwise.

### 2.2. Collection of Epididymal Sperm Samples

After surgery, epididymides and testes were put in zip-lock bags and placed in a styrofoam box. Samples were transported to the laboratory at room temperature (RT) and were processed 3 to 12 h after collection [43,44]. The cauda epididymis was separated from the testis, placed in a tilted Petri dish containing 400 µL of the corresponding cryopreservation medium, depending on the experimental group, and little cuts were performed with a scalpel blade to allow sperm to swim out for 10 min at RT [43,44]. The sperm suspension was transferred to a conical tube (1.5 mL) and sperm parameters were assessed immediately after recovery (step in cryopreservation: T1).

### 2.3. Evaluation of Sperm Traits

The percentage of motile sperm and the quality of movement (using a scale from 0 to 5, being 0 immotile and 5 sperm with vigorous linear forward movement) were evaluated. A sperm motility index (SMI) [(% motile + quality × 20)/2] was calculated as an overall indicator of sperm motility [44,45].

Sperm concentration was assessed with a Neubauer chamber. Sperm were fixed by mixing 5 µL of sperm suspension in 45 µL of 1% glutaraldehyde in cacodylate 0.33 M (pH 7.3) buffer. For the assessments of acrosome integrity and sperm morphology, a 10 µL aliquot was fixed in 250 µL 4% paraformaldehyde. Fixed sperm were stained with Coomassie brilliant blue as described [44].

### 2.4. Sperm Cryopreservation

The effect of two different cryodiluents and two different times of glycerol addition on sperm motility and acrosome integrity were evaluated. The freezing media examined were (a) TEST (*n* = 33) and (b) a Tris-based commercial diluent (Biladyl) (*n* = 32). Their compositions are shown in Table 1.

To evaluate the timing of glycerol addition on sperm quality post-thaw, glycerol was added: (a) in one step before refrigeration or (b) in three steps at the end of refrigeration. When adding glycerol in one step, epididymal sperm were recovered in cryodiluent containing 4% glycerol at RT. Conversely, when adding glycerol in 3 steps, spermatozoa were recovered in cryodiluent with 0% glycerol. At the end of the refrigeration period, cryodiluent containing 8% glycerol was added in 3 steps (each with 1/4, 1/4 and 1/2 of the total final volume, respectively), every 15 min. Thus, if the volume of sperm suspension after refrigerated was 0.5 mL, additions were 125 µL of 8% glycerol, 125 µL of 8% glycerol and, finally, 250 µL of 8% glycerol, achieving a final volume of 1 mL and a concentration of 4% glycerol.

For all treatments, the sperm suspensions were kept in polypropylene tubes (1.5 mL) and cooled in a water bath, using a floating plastic rack. Temperature decreased from room temperature to 5 °C during 120 min (cooling rate: −0.125 °C/min) by adding ice to the bath, with constant monitorization using a digital thermometer.

After refrigeration (step in cryopreservation: T2), samples were taken for sperm motility assessment and aliquots (10 µL) were fixed for acrosome integrity analysis. Subsequently, 50 µL aliquots were loaded in a cold room (at 5 °C) in plastic straws cut in half (0.250 mL original volume) and sealed with a thermal sealer (ERSA, Minitüb, Tiefenbach, Germany). Straws were frozen over nitrogen vapors using a two-level system [44,46] and stored in a liquid nitrogen tank until further analysis.

### 2.5. Sperm Thawing

Frozen samples were transferred from the liquid nitrogen tank to a cryotray containing liquid nitrogen. A standard protocol was used for thawing [43]. Briefly, each straw was exposed to air for 10 s (to eliminate any remaining LN_2_ on the straw surface) and then immersed in a bath containing sterile saline (NaCl 0.9%, *w*/*v*) at 37 °C for 30 s. Straws were dried with tissue paper, the thermo-sealed tip cut and the sperm suspension poured into a prewarmed sterile microvial (1.5 mL).

The thawed sperm suspension was diluted dropwise with 150 µL of Ham’s F-10 (Irvine Scientific, Izasa, Barcelona, Spain) supplemented with 5% (*v*/*v*) inactivated fetal bovine serum (FBS), 0.292 mg L glutamine/mL, 0.110 mg pyruvate/mL and antibiotics (130 IU penicillin/mL, 130 µg streptomycin/mL and 260 µg neomycin/mL).

Sperm quality was examined right after dilution in supplemented Ham’s F10 medium. Motility and acrosome integrity were evaluated as described above (step in cryopreservation: T3). The remaining sperm suspension was incubated for 4.5 h at 37 °C under air. Aliquots were taken for motility and acrosome integrity evaluation at 90 (T4), 150 (T5), 210 (T6) and 270 min (T7) of incubation.

Frozen-thawed sperm samples to be used in IVF assays (see below) were resuspended in a modified Tyrode’s medium (instead of Ham’s F-10), supplemented with 15 mM Hepes, 0.36 mM pyruvate, 2.2 mM calcium lactate, 1 mM L-glutamine, 100 μg penicillin/mL, 100 μg streptomycin/mL and 0.6% (*w*/*v*) BSA (fatty acid free; catalog no. 1265479; Calbiochem, Madrid, Spain) [47,48,49].

### 2.6. Sperm Preparation for IVF: Swim-Up and Pentoxifylline Addition

To examine the potential improvement of sperm quality after thawing, the effect of centrifugation and swim-up was assessed. Frozen–thawed samples were centrifuged at 400× *g* for 8 min. The supernatant was removed and the sperm pellet was resuspended in 100 µL Tyrode medium (without Hepes). The sperm suspension was incubated for 30 min at 38.5 °C (under 5% CO_2_ in air and maximum humidity) to allow motile spermatozoa to migrate into the medium above the pellet. When the incubation ended, an aliquot of 75 µL was recovered from the upper portion of the sperm suspension. Motility was evaluated and an aliquot fixed for the acrosome integrity assessment. The remaining sperm suspension was incubated for 2 h under the same conditions. Motility and acrosome integrity were evaluated at 1 and 2 h of incubation. From each thawed sample, an aliquot was centrifuged and allowed to swim-up and another one was simply diluted and used as control.

To examine the effect of the motility stimulant pentoxifylline, the thawed sperm suspension was divided into two aliquots, both diluted (1:1, *v*/*v*) in Tyrode medium (without Hepes). One of the aliquots was supplemented with pentoxifylline (1 mM final concentration) [39] and the other, with no additions, served as control. Samples for motility and acrosome integrity were obtained. The sperm suspensions were incubated for 1 h at 38.5 °C under 5% CO_2_ in air and maximum humidity and motility and acrosome integrity were re-evaluated after incubation.

### 2.7. Oocyte Recovery and In Vitro Maturation (IVM)

The ovaries from ovariohysterectomized queens (*n* = 72), 6–12 months old, were obtained from local veterinary clinics and transported to the lab at 5 °C in 15 mL vials containing 5 mL of sterile saline (NaCl 0.9%, *w*/*v*) supplemented with 100 µg penicillin/mL and 100 µg streptomycin/mL. The ovaries were processed just after surgery or stored at 5 °C for a maximum of 24 h. Samples were processed as previously described [47], with some modifications [48], and oocytes were incubated for 24 h at 38.5 °C under 5% CO_2_ in air and maximum humidity.

### 2.8. IVF of In Vitro Matured Oocytes with Frozen–Thawed Spermatozoa

After 24 h of in vitro maturation, domestic cat oocytes were co-incubated with frozen-thawed spermatozoa. For fertilization, the oocytes were placed in 50–100 µL drops (10–20 oocytes/drop), in a Petri dish, in a modified Tyrode’s solution with 15 mM NaHCO_3_, 0.36 mM pyruvate, 2.2 mM calcium lactate, 1 mM L-glutamine, 100 µg penicillin/mL, 100 µg streptomycin/mL, and 0.6% (*w*/*v*) BSA (fatty acid free, Calbiochem) [47,48,49]. The gametes were co-incubated for 18–20 h at 38.5 °C with 4 × 10^5^ motile spermatozoa/mL, under mineral oil, 5% CO_2_ in air and maximum humidity.

After gamete co-incubation, oocytes were pipetted gently to remove cumulus cells and unattached spermatozoa and then washed three times using Hepes-buffered Tyrode’s medium. Presumptive zygotes were transferred to four-well culture dishes with 500 μL Tyrode’s solution containing also 1% (*v*/*v*) minimum essential medium (MEM) non-essential amino acids, 0.3% (*w*/*v*) BSA (fatty acid free) and supplemented with 15 mM NaHCO_3_, 0.36 mM pyruvate, 2.2 mM calcium lactate, 1 mM glutamine, 100 μg penicillin/mL and 100 μg streptomycin/mL [47,48,49]. The culture was carried out under 5% CO_2_ in air at 38.5 °C. Cleavage was evaluated 44–48 h after insemination. Non-cleaved oocytes were stained to evaluate meiotic progression and to assess fertilization with Hoechst 33342 (10 μg/mL) in glycerol (1:9, *v*/*v*), mounted onto a slide in the same medium, covered with a coverslip supported by vaseline:paraffin (10:0.7, *w*/*w*) and kept refrigerated at 5 °C in the dark until examination. Both early embryos and oocytes with the presence of pronuclei or decondensed sperm heads were considered as fertilized. The proportion of fertilized oocytes was expressed over metaphase II oocytes as IVF rates.

### 2.9. Statistical Analysis

Statistical analysis was carried out with SPSS v. 11.5 (SPSS Inc., Chicago, IL, USA). Results are presented as mean ± SEM and values of *p* < 0.05 were considered statistically significant. The effects of cryodiluent, time of glycerol addition, swim-up and pentoxifylline supplementation were analyzed by split-plot ANOVA and subsequent multiple comparisons analysis. Relationships between sperm parameters and fertilization success were analyzed by parametric Pearson’s correlation. Fertilization rates for spermatozoa cryopreserved in TEST or Biladyl were compared using the nonparametric Wilcoxon signed-rank test for paired samples.

## 3. Results

### 3.1. Effect of Cryodiluent: TEST vs. Biladyl

**Figure 1:**
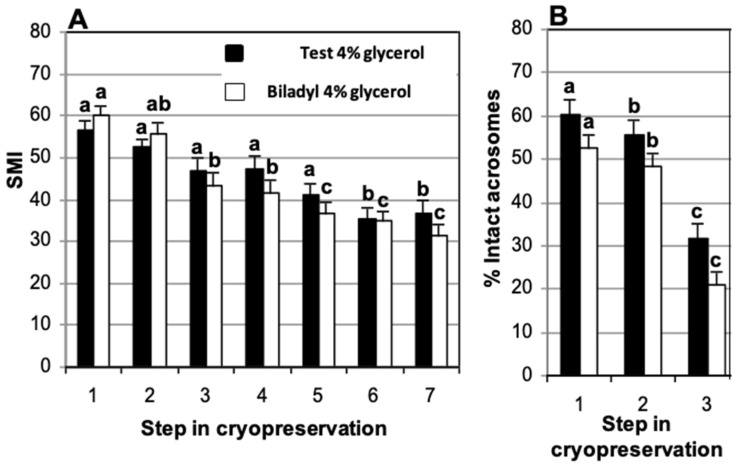
Comparison of cryodiluents (TEST vs. Biladyl) used for domestic cat epididymal sperm. Sperm were suspended in cryodiluent with 4% glycerol, refrigerated at −0.125 °C/min and stored in straws loaded at 5 °C. (**A**) Sperm motility index (SMI), (**B**) percentage of spermatozoa with intact acrosome. The X axis indicates the cryopreservation steps: (1) after recovery (fresh), (2) after refrigeration, (3) after thawing, and (4, 5, 6 and 7) at 90, 150, 210 and 270 min of incubation post-thaw in air at 37 °C. There are no significant differences (*p* > 0.05) between TEST (*n* = 33) and Biladyl (*n* = 32). (a–c) Different letters within diluent indicate significant differences (*p* < 0.05) over time. Analysis was carried out using split-plot ANOVA.

There was an overall decrease in motility in both diluents during the process of cryopreservation and incubation after thawing (Figure 1A). In TEST, no significant difference was observed in motility after thawing (T3) in relation to motility in fresh samples (T1). In contrast, for Biladyl, a significant lower value of SMI was found after thawing (T3) in comparison to fresh samples (T1). After thawing, there was a significant decrease in motility at 210 min of incubation (T6) in TEST in comparison to the value seen after thawing. This significant decrease occurred earlier, at 150 min (T5), in Biladyl. Despite the different patterns seen for each diluent, there were no significant differences in motility between diluents at the different steps of cryopreservation, including the incubation after thawing.

The proportion of spermatozoa with intact acrosomes recorded in fresh samples (60.5 ± 3.1% and 52.5 ± 3.2%, in TEST and Biladyl, respectively) significantly decreased after refrigeration (55.8 ± 3.3% and 48.5 ± 3.0%, respectively) and after thawing (31.7 ± 3.6% and 21.0 ± 2.9%, respectively) (Figure 1B). However, no significant difference was seen between cryodiluents in the percentage of spermatozoa with intact acrosomes (31.7 ± 3.6% and 21.0 ± 2.9%, respectively) after thawing.

### 3.2. Effect of Glycerol Addition System in TEST Cryodiluent: One vs. Three Steps

**Figure 2:**
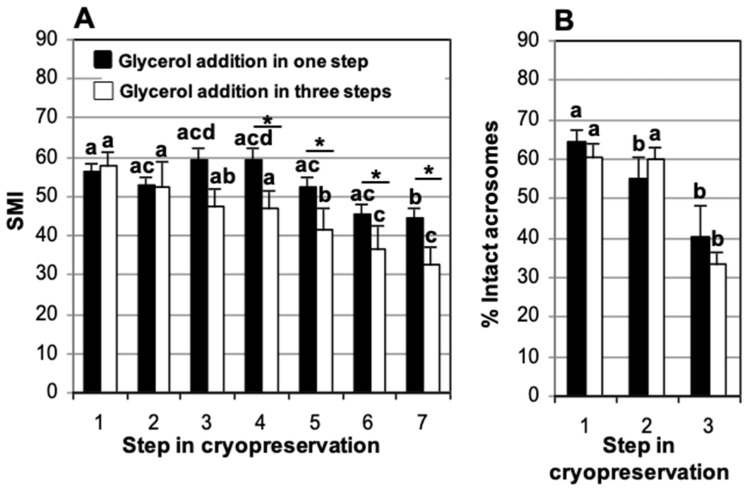
Comparison of the effect of glycerol addition system (one step vs. three steps) on domestic cat epididymal sperm cryopreservation using TEST cryodiluent. Glycerol final concentration was 4%. (**A**) Sperm motility index (SMI) and (**B**) percentage of spermatozoa with intact acrosomes. The X-axis represents the cryopreservation steps: (1) after recovery (fresh), (2) after refrigeration, (3) after thawing, and (4, 5, 6, and 7) at 90, 150, 210, and 270 min of post-thaw incubation in air at 37 °C. An asterisk (*) indicates significant differences (*p* < 0.05) between the two systems (one-step, *n* = 15; three-step, *n* = 10) at each cryopreservation step. (a–d) Different letters within diluent indicate significant differences (*p* < 0.05) over time. Analysis was carried out using split-plot ANOVA.

We analyzed differences in motility and acrosome integrity between samples collected and refrigerated in TEST with 4% glycerol or collected in TEST with 0% glycerol, refrigerated, and with the addition of TEST containing 8% glycerol in three steps (see Materials and Methods for details). All samples were cooled slowly (cooling rate of −0.125 °C/min), loaded into straws at 5 °C, and frozen in two levels over nitrogen vapor. We did not observe significant differences between the two groups, in either SMI or acrosome integrity, at the end of refrigeration or immediately after thawing (Figure 2).

We found significant differences in SMI between the two glycerol addition methods during post-thaw incubation. At 1.5 h of incubation, SMI was 59.4 ± 4.0 and 46.8 ± 4.5, when glycerol was added in one step and three steps, respectively. These differences persisted after 2.5 h incubation (52.2 ± 2.4 and 41.4 ± 5.6, respectively), 3.5 h (45.3 ± 1.6 and 36.4 ± 5.9, respectively), and 4.5 h (44.3 ± 4.0 and 32.5 ± 4.7, respectively) of incubation (Figure 2A). On the other hand, we did not observe a significant decrease in SMI during the cryopreservation process.

A significant decrease was observed in the proportion of spermatozoa with intact acrosomes between samples immediately after sperm recovery (64.4 ± 3.1% and 60.5 ± 2.9% with glycerol added in one or three steps) and post-thawing (40.3 ± 7.8% and 33.3 ± 3.5%, respectively). However, no differences were seen due to the method of glycerol addition (Figure 2B).

### 3.3. Relationships Between Sperm Parameters Before and After Cryopreservation

**Figure 3:**
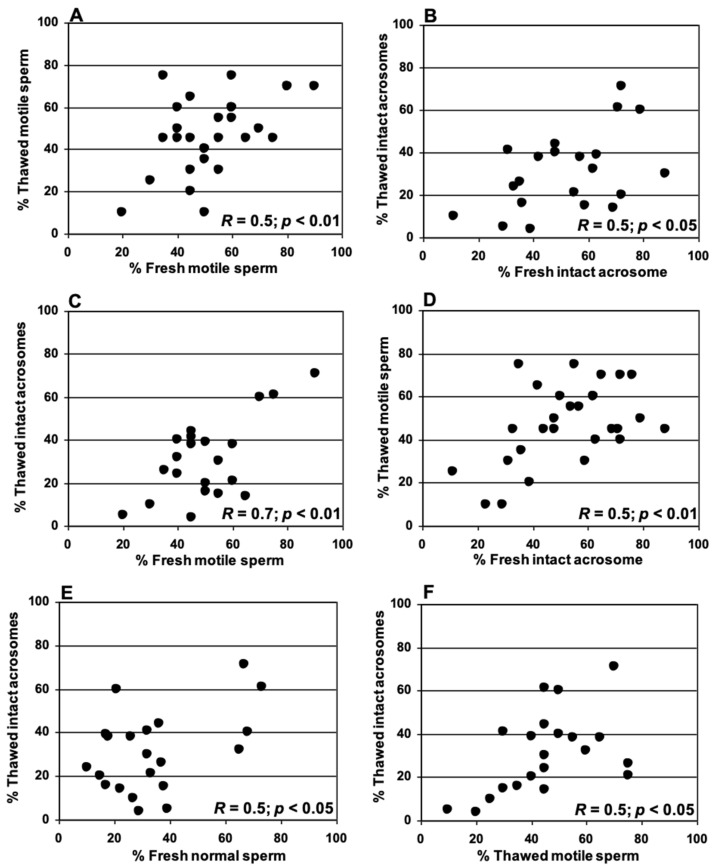
Correlations between sperm parameters (% motile sperm, % spermatozoa with intact acrosomes, and % normal sperm) before (fresh) vs. after (thawed) cryopreservation. (**A**) % motile sperm in fresh and frozen-thawed samples, (**B**) % spermatozoa with intact acrosomes in fresh and frozen-thawed samples, (**C**) % motile sperm in fresh samples and % spermatozoa with intact acrosomes after thawing, (**D**) % spermatozoa with intact acrosomes in fresh samples and % motile sperm after thawing, (**E**) % normal sperm in fresh samples and % spermatozoa with intact acrosomes after thawing, (**F**) % motile sperm and % spermatozoa with intact acrosomes after thawing. *R* is the Pearson correlation coefficient, and *p* is the associated critical level.

Sperm parameters in samples collected and cryopreserved in TEST or Biladyl with glycerol added in one step were studied. Significant positive correlations between the proportion of motile sperm (Figure 3A) and the proportion of spermatozoa with intact acrosomes (Figure 3B) in samples before (fresh) and after (thawed) cryopreservation were detected. Furthermore, we found a significant positive correlation between the proportion of motile spermatozoa in fresh samples and the proportion of intact acrosomes in thawed samples (Figure 3C), and between the proportion of intact acrosomes in fresh samples and the proportion of motile spermatozoa after thawing (Figure 3D).

Additionally, a significant positive correlation between the proportion of normal spermatozoa in fresh samples and the proportion of intact acrosomes appeared after thawing (Figure 3E). Finally, we found a significant correlation between the proportions of motile sperm and spermatozoa with intact acrosomes after thawing (Figure 3F).

### 3.4. Sperm Preparation for IVF

**Figure 4:**
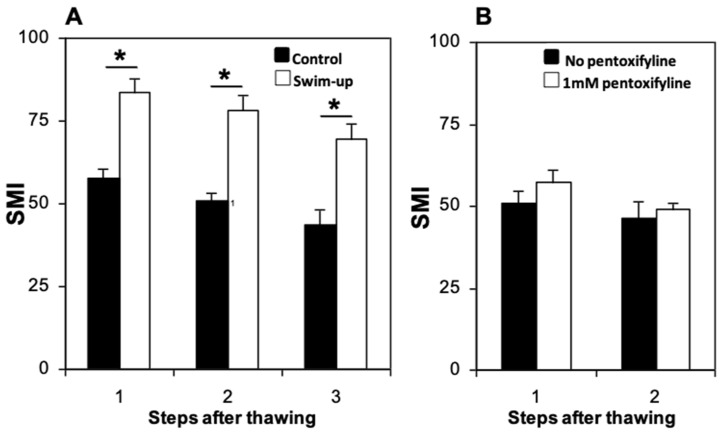
Effect of centrifugation and swim-up or addition of pentoxifylline after thawing on domestic cat epididymal sperm motility. (**A**) Effect of centrifugation. (**B**) Effect of addition of 1 mM pentoxifylline. Steps after thawing were: 1, immediately after thawing; 2, 1 h after thawing; 3, 2 h after thawing. At each step after thawing, an asterisk (*) indicates significant differences (*p* < 0.05) between control and treatment. Analysis was carried out using split-plot ANOVA. SMI: Sperm motility index.

The preparation of sperm by using swim-up after thawing resulted in SMI values (from 83.8 ± 3.81 just after thawing, to 69.4 ± 4.7 after incubation for 2 h) that were significantly higher than those found in samples that were simply diluted after thawing (from 57.9 ± 2.5 just after thawing, to 43.8 ± 4.6 after 2 h) (Figure 4). No significant differences were observed in the proportion of intact acrosomes between samples allowed to swim-up and those that were diluted There were no significant changes over time in either the SMI (Figure 4) or in the percentage of intact acrosomes.

No significant differences were observed in SMI between spermatozoa incubated with 1 mM pentoxifylline and untreated controls, both immediately after thawing (57.5 ± 3.7 and 50.7 ± 4.0, respectively) and 1 h after incubation post-thaw (48.9 ± 1.8 and 46.4 ± 4.9, respectively) (Figure 4B). The percentage of spermatozoa with intact acrosomes was similar between the two groups (with or without pentoxifylline) just after thawing (40.4 ± 6.8% and 39.4 ± 8.1%, respectively), and 1 h after incubation post-thaw (31.3 ± 7.5% and 29.9 ± 7.0%, respectively). Comparisons of sperm samples over time revealed no significant decreases in SMI (Figure 4B) or in percentage of intact acrosomes. Thus, the supplementation of the thawing medium with 1 mM pentoxifylline did not result in an improvement of sperm quality over the control without pentoxifylline.

### 3.5. IVF of In Vitro Matured Oocytes with Frozen–Thawed Domestic Cat Epididymal Spermatozoa

We evaluated the fertilization capacity of cryopreserved spermatozoa by IVF using frozen–thawed spermatozoa. The sperm samples used (*n* = 3 replicates each) were those used in experiments comparing cryodiluents (TEST vs. Biladyl) and glycerol addition (one step vs. three steps in TEST cryodiluent). Control samples (fresh spermatozoa), recovered in Hepes-buffered Tyrode medium, were used as controls.

Spermatozoa cryopreserved in TEST with glycerol added in one step fertilized 33.3 ± 8.5% of oocytes, which was not significantly different from the fertilization rate observed using fresh spermatozoa (49.0 ± 8.6%). There were no significant differences between the fertilization rates obtained with frozen–thawed spermatozoa in TEST with glycerol added in one step or three steps (26.2 ± 14.5%).

**Figure 5:**
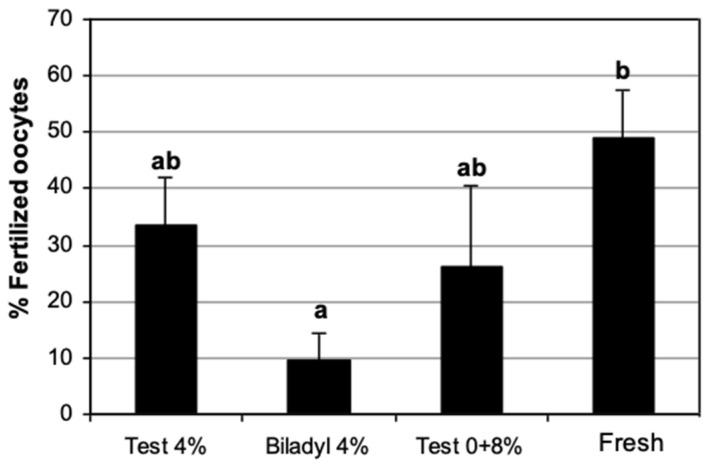
IVF of domestic cat in vitro matured oocytes with cat epididymal spermatozoa cryopreserved in two diluents (TEST vs. Biladyl) using two glycerol addition methods (one-step [4%] vs. three-step [0 + 8%]) or freshly collected in Hepes-Tyrode’s medium (control group). (a,b) Different letters indicate significant differences (*p* < 0.05) between treatments (*n* = 3 each). Analysis was carried out using split-plot ANOVA.

When spermatozoa were cryopreserved in Biladyl, the fertilization rate obtained (9.5 ± 4.7%) was significantly lower than that achieved using fresh spermatozoa. There were no significant differences between spermatozoa cryopreserved in Biladyl and those cryopreserved in TEST with glycerol added in one or three steps (Figure 5).

We found no correlation between the IVF rate and the percentage of motile, intact acrosome, and normal spermatozoa in fresh or thawed samples.

The treatments used to prepare the spermatozoa after thawing did not significantly influence the IVF rate. Despite the significant differences obtained in motility (SMI) between swim-up selected spermatozoa and controls, we did not observe significant differences in the IVF rate (% fertilized oocytes) using sperm collected by swim-up (37.8 ± 10.4%) or with sperm samples simply diluted (36.0 ± 8.4%) after thawing.

In another experimental series, we analyzed the fertilization ability of spermatozoa in connection with pentoxifylline addition. We obtained 20.7 ± 11.4% (range: 0.0–83.3%) of fertilization when spermatozoa were exposed to thawing medium with 1 mM pentoxifylline and 20.5 ± 8.1% (range: 0.0–57.1%) in the control group, which agrees with the lack of significant differences found in sperm motility.

## 4. Discussion

The results of this study have shed light on the cryopreservation of domestic cat epididymal sperm, analyzing the effect of two key factors (cryodiluent and timing of glycerol addition) on motility, acrosome integrity, and the fertilization capacity of cryopreserved spermatozoa using IVF of in vitro matured domestic cat oocytes.

The sperm motility index (SMI) and the percentage of spermatozoa with intact acrosomes were similar between TEST and Biladyl. A sharper decrease in SMI occurred in samples cryopreserved in Biladyl compared to those in TEST. This agrees with previous reports of a sharp decline in motility and morphologically normal sperm when using a Tris-citric acid-fructose diluent (similar to Biladyl) for the cryopreservation of epididymal cat sperm [25]. In contrast, studies in sheep [50], goats [51] and bulls [52] found greater post-thaw sperm motility when semen was diluted with Biladyl. These discrepancies may be due to differences in the species (ungulates vs. felids) and the origin of the sperm (ejaculated vs. epididymis). We conclude that TEST is superior to Biladyl for freezing domestic cat epididymal spermatozoa, as it maintains higher motility values for a longer period. Given the composition of both cryodiluents, it is possible that a buffer system based on Tes-Tris (as seen in TEST cryodiluent) may sustain sperm motility more effectively than a buffer system based in Tris-citric acid (as in Biladyl). In addition, differences in sugar composition (glucose in TEST vs. fructose in Biladyl) may exert some effect in sperm survival and motility, although this may be unlikely for several reasons. For example, no differences were observed when glucose or fructose were added to cat cryodiluents [25]. Furthermore, the essential glycolytic activity of cat sperm seems to be unrelated to hexose metabolism [53].

Other studies have examined the effect of cryodiluents on domestic cat epididymal sperm cryopreservation, comparing commercial test yolk buffer with a lactose-EDTA- based cryodiluent, and have observed better results for motile, viable, and morphologically normal spermatozoa after thawing with the latter [54]. Results obtained with ejaculated spermatozoa from tigers (*Panthera tigris*) suggest a similar conclusion [55,56]. Additional studies have investigated the effect of various additives, such as carnitine, which is an antioxidant that improves energy production in mitochondria and protects DNA and sperm membrane from ROS-induced damage [57] and Equex, a detergent that enhances the protective function of egg yolk [33,58], which also improve sperm quality. Further research is needed to clarify the preference for one cryodiluent over another when cryopreserving domestic or wild cat spermatozoa.

Although the gradual addition of the cryoprotectant has been suggested to minimize the loss of motility and membrane disruption [23], we found no significant differences in either motility or acrosome integrity up to 1.5 h after thawing between samples with glycerol added in one step (before refrigeration) or three steps (at the end of refrigeration). However, after 1.5 h incubation post-thaw, significant differences in SMI were detected, with spermatozoa exposed to glycerol in one step at room temperature showing significantly better motility than those exposed to glycerol in three steps at 5 °C, in accordance with previous reports in domestic cats [5]. Other studies found improved motility and longevity of post-thawed cat sperm [32], and greater motility and acrosome integrity in fishing cats (*Prionailurus viverrinus*) when glycerol was added at 5 °C instead of 25 °C [59]. This discrepancy could be attributed to: (1) the gradual glycerol addition in the fishing cat study, regardless of temperature, and (2) the use of epididymal spermatozoa in our study versus ejaculated spermatozoa in the fishing cat study [38]. In any case, some studies have found no significant differences in cryosensitivity between these two types of spermatozoa [60,61].

The significant positive correlations found between motility and acrosome integrity before and after cryopreservation support the value of these parameters as indicators of cryosurvival. Since teratospermia is common among a wide range of felids [62], we assessed morphological abnormalities in fresh samples to test whether the prevalence of abnormal sperm relates to post-thaw parameters. The correlations between the proportion of normal sperm in fresh samples and the proportion of intact acrosomes in thawed samples highlight the relationship between morphology and cryosensitivity, supporting the importance of morphology and structural integrity as key indicators of sperm cryosurvival [46]. These findings are consistent with other studies that reported a positive correlation between the percentage of normal sperm and the percentage of spermatozoa with intact acrosomes [63,64]. However, they differ from those obtained in other studies on domestic cat epididymal sperm, where no significant correlation between the percentage of normal sperm in fresh samples and the percentage of intact acrosomes after thawing were observed [65].

Given the reduction in motility and acrosome integrity caused by sperm cryopreservation [66], we analyzed the effect of swim-up to select spermatozoa with greater motility in frozen–thawed samples. It has been reported [53] that sperm recovered after swim-up showed improved motility, lactate production and pyruvate uptake compared to unprocessed sperm. In our study, swim-up proved to be a very effective method of sperm selection as SMI significantly increased in sperm samples allowed to swim up immediately after thawing. However, this method substantially reduces sperm concentration, which can be critical in feline species that produce a small total number of spermatozoa per ejaculate [67].

On the other hand, the lack of a significant effect from 1 mM pentoxifylline added to the thawing medium could be due to the concentration used, as the effect of this and other motility stimulants are dose-dependent [39]. It has been reported that a minimum concentration is necessary for pentoxifylline to enhance sperm motility [39,68]. On the other hand, excessive concentrations and prolonged exposure may negatively impact sperm parameters and membrane integrity. Previous studies evaluating the effect of several motility stimulants after cryopreservation described improvements in cat sperm motion by pentoxifylline [39,69]. In those studies, both epididymal and ejaculated cat spermatozoa were treated with pentoxifylline for 15 min. This brief treatment was sufficient to increase the percentage of motile sperm, curvilinear velocity and the amplitude of head lateral displacement compared to untreated sperm, as assessed by CASA, without compromising sperm longevity [39,69]. In addition, using a salt-stored egg penetration assay, the addition of pentoxifylline to snow leopard spermatozoa enhanced their ability to bind to the zona pellucida of domestic cat oocytes [70]. Pentoxifylline also improved the SMI but was unable to maintain a higher SMI over time compared to untreated samples in snow leopard sperm [70]. Nevertheless, the acrosome integrity values analyzed in our study represent the first evaluation of this parameter in domestic cat spermatozoa stimulated with pentoxifylline and will be useful for evaluating the effect of phosphodiesterase inhibitors on acrosome reaction and sperm capacitation [38].

Although cryopreservation has been reported to cause alterations in sperm motility, morphology, and structural integrity after thawing, and consequently, to reduce sperm fertilizing capacity [10], we only observed a significant reduction in IVF rates, compared to fresh spermatozoa, when Biladyl with glycerol added in one step was used. Therefore, TEST seems to be adequate as a cryodiluent for domestic cat epididymal spermatozoa.

No significant correlations were found in our study between the percentage of fertilized oocytes and the percentage of motile, intact acrosomes, or normal spermatozoa. These results differ from previous findings, which reported a significant correlation between post-thaw SMI and oocyte penetration rate in domestic cats [14]. One possible explanation for this discrepancy is the source of spermatozoa ejaculated [14] vs. epididymal (our study). Components of seminal plasma may alter the sperm during and after ejaculation in such a way that a different response is elicited. It is also worth noting that in conventional IVF systems, variables such as the incubation period of gametes and the number of spermatozoa interacting with oocytes may affect IVF outcomes. These parameters are usually optimized to maximize embryo production. Thus, in order to detect differences due to sperm quality, a reduced concentration of spermatozoa and/or a limited time of gamete co-incubation may be needed, so that an excess of male gametes or prolonged interaction does not compensate for differences in fertilizing ability [71,72].

## 5. Conclusions

The results obtained in this work showed that, in domestic cat epididymal sperm cryopreservation: (1) the use of TEST or Biladyl did not result in major differences in sperm motility nor in the proportion of spermatozoa with intact acrosomes after thawing; (2) the addition of glycerol in one or three steps when using the TEST cryodiluent did not result in differences in motility or acrosome integrity after thawing; (3) motility, acrosome integrity, and normal morphology covaried throughout the cryopreservation process and may serve as suitable indicators of sperm cryosurvival; (4) swim-up enhanced motility but not the IVF rate of cryopreserved spermatozoa; and (5) cryopreservation in TEST led to fertilization rates similar to those obtained using fresh spermatozoa.

## Figures and Tables

**Table 1:** Composition of cryodiluents used for cat sperm cryopreservation: TEST and Biladyl.

Component	TEST	Biladyl
Tes	4.83%	-
Tris	1.15%	2.42%
Glucose	0.4%	-
Fructose	-	1.00%
Citric Acid	-	1.38%
Egg yolk	20%	20%
Glycerol (*)	4%	4%
Penicillin (IU/mL)	200	200
Streptomycin (µg/mL)	200	200
pH	7.2	7.0
Osmolarity (mOsm/L)	360	340

(*) The final glycerol concentration was 4%. Both diluents, TEST and Biladyl, were prepared with glycerol concentrations of 0%, 4% and 8%. Addition of the diluent was performed in 1 step, before refrigeration, with 4% glycerol, or in 3 steps after refrigeration. Osmolarity was measured before the addition of glycerol. Osmolality was measured using a freezing point depression osmometer.

## Data Availability

The original contributions presented in this study are included in the article. Further inquiries can be directed to the corresponding author.
